# Molecular cloning and development of RAPD-SCAR markers for *Dimocarpus longan* variety authentication

**DOI:** 10.1186/2193-1801-2-501

**Published:** 2013-10-03

**Authors:** Luquan Yang, Shelly Fu, Md Asaduzzaman Khan, Weimin Zeng, Junjiang Fu

**Affiliations:** Research Center for Preclinical Medicine, Luzhou Medical College, Luzhou, Sichuan 646000 China; Michael E. DeBakey High School for Health Professions, 3100 Shenandoah Street, Houston, TX 77021 USA; Department of Biochemistry, School of Life Sciences, Central South University, Changsha, Hunan 410013 China

**Keywords:** *Dimocarpus longan* Lour, *Dimocarpus confinis*, Improved RAPD, Cloning, SCAR marker, Authentication

## Abstract

As an edible fruit and source of traditional medicine, *D. longan* is grown in most areas of Southern China. Identification of *D. longan* cultivars by using molecular markers is important genetically. In this study, we cloned fragments from improved randomly amplified polymorphic DNA (RAPD), and developed stably diagnostic sequence-characterized amplified region (SCAR) markers. The specific RAPD bands of *D. longan* cultivars from Guangxi, with size ranging from 500 bp to 900 bp were gel-purified, cloned and sequenced. Four clones named LY2-1, LY4-7, LY4-8 and LY5-2 were identified. In order to investigate whether the fragments were specific for the species, four pairs of SCAR primers were then designed. PCR amplifications were conducted to analyze 18 samples including different *D. longan* cultivars and other species. The specific bands with expected sizes were amplified in five *D. longan* samples but not in others. To identify and characterize the difference between *D. longan* and *D. confinis*, PCR amplifications were performed again. The specific bands with expected sizes were found in *D. longan* but not in *D. confinis* by SCAR markers LY2-1, LY4-7 and LY5-2, respectively. These results showed that our developed SCAR markers could be very useful as a specific *D. longan* variety authentication. Therefore, our study provides an effective and precise PCR-based diagnostic method and markers to identify *D. longan* species.

## Introduction

*Dimocarpus longan* Lour (*D. longan*), also called longan or dragon eye, is a commercially attractive fruit that is widely distributed in subtropical areas. China is the world’s largest cultivator of *D. longan*, with more than 2000 years history of cultivation. As a traditional medicine, *D. longan* fruit is used for enhancing memory, promoting blood metabolism, relieving insomnia, and preventing amnesia. Its secondary metabolic products have been also shown to have anti-oxidative, anti-obesity, anti-cancer, anti-tyrosinase, and immunomodulatory activities (Park et al. 2010; Prasad et al. 2010; Zhong et al. 2010). A number of researches have been conducted to give an insight of genetic characterization and authentication in *D. longan* samples.

Several molecular markers have been developed and applied since 1990, including random amplified polymorphic DNA (RAPD) (Williams et al. 1990; Devaiah and Venkatasubramanian, 2008; Chen et al. 2010; Yazbeck et al. 2011; Bhat et al. 2012; Shakeel et al. 2013; Noormohammadi et al. 2013; Zhang et al. 2013), inter-simple sequence repeats (Feofilov et al. 2011; Ganopoulos et al. 2011; Noormohammadi et al. 2013; Zhang et al. 2013), internal transcribed spaces (Varela et al. 2004) and amplified fragment length polymorphism (Vos et al. 1995). These molecular markers have been extensively utilized in various fields for the assessment of genetic diversity, genotype fingerprinting, and molecular breeding.

RAPD markers are DNA fragments from PCR amplification of the genomic DNA’s random segments with single primer of arbitrary nucleotide sequence (Williams et al. 1990). It is a relatively easy, inexpensive and rapid technique because of its simplicity and requirement for minimal amounts of genomic DNA (Micheli et al. 1994). It has been widely used in the identification and genetic relationship analysis of a number of plant and animal species. The improved RAPD can improve the resolution of the PCR products and its repeatability (Fu et al. 2000; Fu et al. 2013; Mei et al. 2013). The sequence characterized amplified region (SCAR) marker is one of the stable markers, generally derived from random amplified polymorphic DNA (RAPD) (Dnyaneshwar et al. 2006; Li et al. 2010; Rajesh et al. 2013). The basic principle is to convert the dominant markers into co-dominant markers to reduce the tedious procedures of RAPD (Li et al. 2010; Rajesh et al. 2013). These markers generally reveal higher levels of polymorphism owing to higher annealing temperatures and longer primer sequence specificity (Kumla, et al. 2012). With SCAR marker, analysis is reduced to a simple PCR analysis using PCR primers designed from the sequence of the amplicon of RAPD (Kumla, et al. 2012; Rajesh et al. 2013).

*Dimocarpus confinis* is a species of plant in the genus Dimocarpus, which is grown in a range of Southern China to Southeast Asia. It is mainly grown as ornamental plants but cannot be used as a food source due to its poisonous nature. *D. confinis* produces oval-shaped drupe fruits, which is very similar to *D. longan*, causing difficulty in distinguishing it from *D. longan* in the market, if anyone ever sells *D. confinis* as longan [Source: http://www.people.com.cn/GB/paper503/13144/1179090.html]. In this study, we aimed to distinguish *D. longan* from *D. confinis* by genetic characterization using RAPD and SCAR. In order to increase the reproducibility and reliability of PCR assays in *D. longan* and distinguish it from *D. confinis*, SCAR markers have been developed from clones of RAPD fragments. In the present study, the DNA fragments were amplified with the DNA template of *D. longan* from Guangxi Province, using an improved RAPD (Fu et al. 2000; Fu et al. 2013; Mei et al. 2013) followed by DNA ligation, cloning, and sequencing. After a series of experiments, four new specific longan DNA fragments had been confirmed. According to the sequencing results, 4 pairs of primers (SCAR markers) had been designed to converted, which detected specificity of *D. longan* varieties. Three of SCAR markers were useful to distinguish *D. confinis* from *D. longan*. Therefore, the SCAR primers can be used to assess the genetic diversity and population structure of *D. longan* from *D. confinis*.

## Materials and methods

### Genomic DNA extraction

The DNAs were extracted from fresh young leaves of *D. longan* and *D. confinis* by using previously described slightly modified Cetyl trimethylammonium bromide (CTAB) method (Mei et al. 2013) and stored at -20°C till to use. The fresh young leaves of *D. confinis* were provided by Dr. Jiechun Pan from Agricultural College of Guangxi University in Guangxi Province. Leaves were first fixed in fixing solutions containing chloroform, (without liquid nitrogen), and then grinded into tiny pieces by silica (SiO_2_) for the extraction of DNA with CTAB method. DNA quality was determined after electrophoresis on 1% agarose gels. DNA concentration and quality was measured by spectrophotometry at 260 and 280 nm and normalized to a concentration of 10 ng/μl, then stored at -20°C for further study (Mei et al. 2013; Fu, 2012).

### Improved RAPD amplification

The *D. longan* DNAs were initially screened with three random primers (Mei et al. 2013). The PCR reactions were performed with Tiangen reagents (Beijing, China). The improved RAPD reaction solution consisted of 7.5 μl 2 × Taq PCR MasterMix, 1.5 μl 2.5 μM primer and 1.5 μl genomic DNA, to a total volume of 15 μl. Amplification reactions were performed in an Eppendorf Authorized Thermal Cycler (Mastercycler 5331 system, Eppendorf, Germany) under the following program, which involves an initial pre denaturation at 95°C for 90 s. It was then followed by 40 cycles of denaturation at 94°C for 40 s, annealing at 36°C for 60 s, and extension at 72°C for 90 s. The final extension step was performed at 72°C for 5 min. During the procedure, the temperature rose at the rate of 0.3°C /s, and declined at the rate of 3°C /s. The amplified products were detected with electrophoresis on 1.5% agarose gel.

### Cloning and sequencing of DNA fragments

Four different bright bands were excised from agarose gel and purified with TIANgel Midi Purification Kit (DP209, China) according to the company provided protocol. Purified DNA fragments were ligated into pGM-T vector (No. VT202) (Tiangen reagents, Beijing, China), and transformed in DH5α *E. coli* complement cells and the recombinant clones were selected on LB agar plates containing 100 μg/μl of ampicillin, 40 mg of X-gal and 160 μg of IPTG. The blue white screening was adopted to find white colony firstly. Then presence of the appropriate insert was verified by PCR with T7/SP6 primer pairs (T7 primer: 5′- TAA TACGACTCACTATAGGG -3′, SP6 primer: 5′-ATTTAGGTGACACTATAGAA-3′), or *EcoR*I digestion, which is located at pGM-T vector nearly to the ligation ends, and then for DNA sequencing (Fu, 2012).

### Sequence homology searches and bio-informatics analysis

Homology searches were performed by online program BLAST from NCBI (http://www.ncbi.nlm.nih.gov/BLAST/) in different species.

### SCAR analysis

The nucleotide sequence of each of the cloned RAPD fragment was used to design pairs of SCAR primers using Primer 3 software from the website (http://bioinfo.ut.ee/primer3-0.4.0/primer3/). Sequences of the SCAR primers, amplification length and PCR condition were shown in Table 1. Eighteen of DNA samples, including eight of *Canavium album* strains, five of *D. longan* varieties LZ, GD, GX, HN, FJ and other 5 kinds of species, which were *Viola. philippica* (DD), *Penthorum sedoides* (GJT), *Penthorum Chinese* (GHC), represents *Lonicera japonica* (JYH), *Gastrodia elata* (TM), were used as templates for PCR amplification and development of SCAR markers. The PCR reaction solution consisted of 7.5 μl 2 × Taq PCR MasterMix, 1.5 μl of 2.5 μM each pair of SCAR primers and 1.5 μl genomic DNA (15 ng), to a total volume of 15 μl. Amplification reactions were performed in an Eppendorf Authorized Thermal cycler with an initial pre-denaturation for 90 s at 95°C followed by 35 cycles of denaturation at 94°C for 40 s, annealing at 60°C for 30 s, and extension at 72°C for 40 s. The final extension step was performed at 72°C for 5 min. The amplified PCR products were resolved by electrophoresis on 1.0% agarose gel in 1 × TAE buffer. Gels were visualized by 0.5 μg/mL ethidium bromide staining and the images were documented using the ChemiDoc XRS(Bio-Rad, USA).

**Table 1:** **Sequences of SCAR primers, PCR condition and product size**

SCAR	5′-primer	Sequence (5′-3′)	3′-primer	Sequence (5′-3′)	Size (bp)	Tm (°C)
LY2-1	LY2-1 L	AACTGGAAGTCCCTGGTCCT	LY2-1R	ACAAGAGGCCCCAGTAAGGT	350	60
LY4-7	LY4-7 L	GGCGCCGGTATACTTTGTAA	LY4-7R	CTCGTAAGAGGATCCGTCCA	367	60
LY4-8	LY4-8 L	CCCCATCTGGTTGTAGCACT	LY4-8R	AGCCAGCTCAACCAACTCAT	358	60
LY5-2	LY5-2 L	TTTTAATGTTGGGCATTTGG	LY5-2R	GCTAACCGAGATCCACTAACG	250	60

To distinguish the difference between *D. longan* and *D. confinis*, PCR amplifications were performed by using above mentioned 4 pairs of SCAR primers and amplification conditions (Table 1).

## Results

### Cloning of RAPD amplification fragments

**Figure 1:**
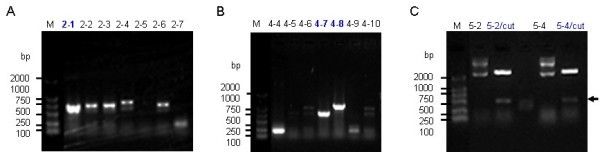
**Cloning and identification of positive clones from** ***D. longan*** **RAPD products. A**. Identification of positive clone 2–1 by PCR amplification with vector T7/sp6 primers. **B**. Identification of positive clones 4–7 and 4–8 by PCR amplification vector T7/sp6 primers. **C**. Identification of positive clones 5–2 and 5–4 by *EcoRI* digestion with extracted plasmids. Lane “M” indicates the DNA molecular weight marker DL2000 with the fragment size (bp) 2000, 1000, 750, 500, 250, 100. The arrow indicates the inserted band in the clones 5–2 and 5–4.

Three RAPD primers (SBS-I4, SBS-Q12, and SBS-Q19) were initially screened using DNA samples from Guangxi *D. longan* (Mei et al. 2013). The characteristic DNA fragments with clear and polymorphic profiles were purified, ligated to T-vector by AT cloning. The blue and white screening method was adopted at first to screen positive cloning. The positive clones 2–1, 4–7, 4–8, 5–2 and 5–4 were identified by PCR amplification using SP6 primer and T7 primer (Figure 1 A and B) or by plasmid DNA digestion using *EcoR*I enzyme (Figure 1C). In the Figure 1A, five clones are positive, but only clone 2–1 showed strongest PCR band with a similar inserted fragment and was selected for further sequencing, whereas in the Figure 1B, clones 4–4, 4–7 and 4–8 are PCR positive, and 4–7 and 4–8 with inserted fragments ~600, ~900 bp DNA-fragment respectively. In the Figure 1C, clones 5–2 and 5–4 with a same ~500 bp inserted fragment digested by *EcoR*I, and clone 5–2 was selected for further sequencing.

### Sequencing and characterization of *D. longan*-specific RAPD fragments

**Figure 2:**
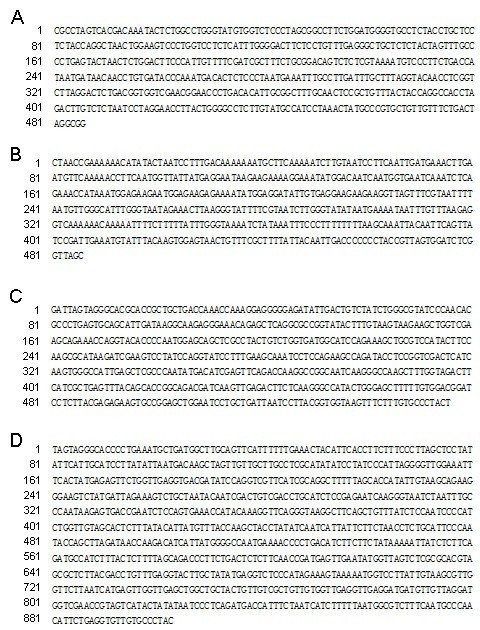
**Cloned sequences information by Sanger-sequencing. A**. The sequences of clone 2–1. **B**. The sequences of clone 5–2. **C**. The sequences of clone 4–7. **D**. The sequences of clone 4–8.

Sequencing of above four cloned RAPD fragments in *D. longan* showed that clone 2–1 consisted of 486 nucleotides and deposited into GenBank with accession number KC522607 (Figure 2A), clone 5–2 consisted of 486 nucleotides and deposited into GenBank with accession number KC522608 (Figure 2B), clone 4–7 consisted of 556 nucleotides and deposited into GenBank with accession number KC522609 (Figure 2C), clone 4–8 consisted of 903 nucleotides and deposited into GenBank with accession number KC522610 (Figure 2D) (Note: the sequences information will not release till one year deposition in Genbank).

**Figure 3:**
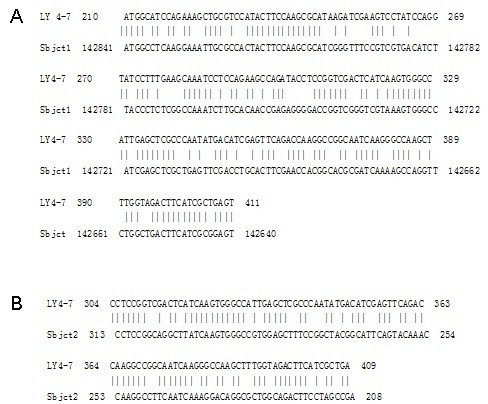
**BLAST searches of the cloned nucleotide sequences.** Showed identity of clone 4–7 with the *Oryza sativa* Japonica Group chromosome 10 clone **(A)**, and with the *Silene vulgaris* isolate 19 J19 retrotransposon putative Retand type sequence **(B)**.

BLAST searches of the nucleotide sequences in GenBank showed that 202 nucleotides of clone 4–7 fragment (nucleotides 210 to 411) shared 67% identity to the *Oryza sativa* Japonica Group chromosome 10 clone OSJNBa0095J15 sequence (Sequence ID: gb|AC092173.3|) with an E value 8e-08 (Figure 3A), and 78 nucleotides in the same region (nucleotides 304 to 409) shared 74% identity to the *Silene vulgaris* isolate 19 J19 retrotransposon putative Retand type sequence (Sequence ID: gb|JN624421.1|), with an E value 3e-07 (Figure 3B). Sequences from other 3 clones didn’t show any significant identity or similarity to any species.

### Development of *D. longan*-specific SCAR markers

**Figure 4:**
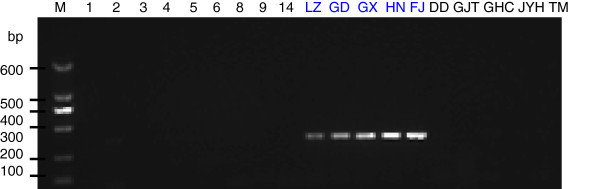
**Analysis of the PCR amplicons of a SCAR marker LY2-1.** 1, 2, 3, 4, 5, 6, 8, 9 and 14 are *C. album* samples collected from Sichuan Province. The samples LZ, GD, GX, HN, FJ were collected from Luzhou in Sichuan, Guangdong, Guangxi, Hainan and Fujian sources of *D. longan* varieties. DD is *V. philippica*. GJT is *P*. *sedoides.* GHC is *P. Chinese* collected from Gulin County in Sichuan Province. JYH is *L. Japonica*; TM is *Gastrodia elata* collected from Liangshan City in Sichuan Province. Lane “M” indicates the DNA molecular weight marker DL600 with the fragment size (bp) 600, 500, 400, 300, 200 and 100.

**Figure 5:**
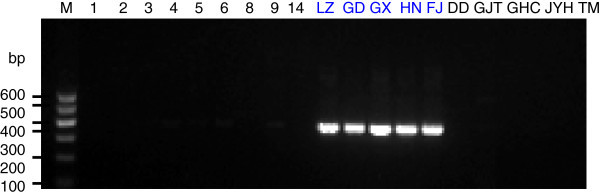
**Analysis of the PCR amplicons of a SCAR marker LY 4–7.** The sample origins and orders indicate on the Figure 4. Lane “M” indicates the DNA molecular weight marker DL600.

**Figure 6:**
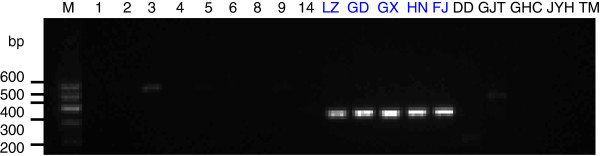
**Analysis of the PCR amplicons of a SCAR marker LY 4–8.** The sample origins and orders indicate on the Figure 4. Lane “M” indicates the DNA molecular weight marker DL600.

**Figure 7:**
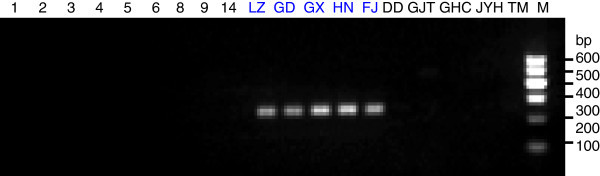
**Analysis of the PCR amplicons of a SCAR marker LY 5–2.** The sample origins and orders indicate on the Figure 4. Lane “M” indicates the DNA molecular weight marker DL600.

To generate stable longan-specific diagnostic SCAR markers from RAPD markers, four pairs of primers (LY2-1 L and LY2-1R; LY4-7 L and LY4-7R; LY4-8 L and LY4-8R; LY5-2 L and LY5-2R) (Table 1) were designed and synthesized based on cloned sequences in Figure 2. The designed SCAR primer pairs were then used to amplify the genomic DNA from 18 of collected DNA samples to test the amplification species-specificity. PCR results indicated that the PCR products with expected size were observed only in five *D. longan* samples by SCAR marker LY2-1 (Figure 4), SCAR marker LY4-7 (Figure 5), SCAR marker LY4-8 (Figure 6), and SCAR marker LY5-2 (Figure 7), without any amplification in other species we tested (Figures 4,5,6,7), which indicated that all four SCAR markers are longan-specific. The lack of this specific amplicon in the other species indicated the efficacy of these marker in distinguishing the longan group from the others.

### Authentication of *D. longan* from *D. confinis*

**Figure 8:**
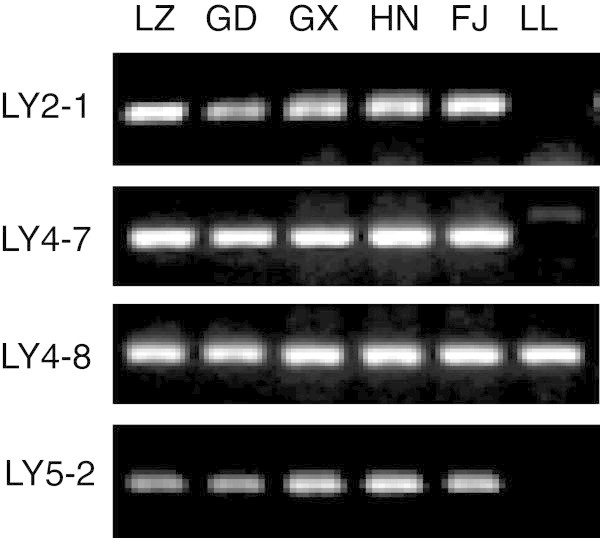
**Identification of** ***D. longan*** **from** ***D. confinis*** **by longan-specific SCAR markers.** The samples LZ, GD, GX, HN, FJ were collected from Luzhou in Sichuan, Guangdong, Guangxi, Hainan and Fujian sources of *D. longan*. The sample LL was collected from Guangxi source of wild *D. confinis*. LY2-1, LY4-7, LY4-8 and LY5-2 represent longan-specific SCAR markers for LY2-1, LY4-7, LY4-8 and LY5-2 which were used in the PCR amplification, respectively.

To distinguish *D. longan* from *D. confinis*, PCR amplification were performed by using our developed four pairs of SCAR primers (Table 1) with conditions mentioned in Material and Methods. The result showed that the PCR products with expected size were observed only in five *D. longan* samples by SCAR markers LY2-1, LY4-7 and LY5-2, without any amplification in the sample of *D. confinis*, which indicated that these three makers are useful for the identification *D. longan* from *D. confinis* (Figure 8). However, we still noticed the PCR product in *D. confinis* (LL) with same expected size from *D. longan* by SCAR marker LY4-8, which indicated that this marker can’t be used in the authentication of *D. longan* from *D. confinis* (Figure 8)*.*

## Discussion

RAPD analysis can reveal high degrees of polymorphisms, does not require prior DNA sequence information of the species, and is easy to manipulate (Williams et al. 1990; Devaiah and Venkatasubramanian, 2008; Chen et al. 2010; Yazbeck et al. 2011; Bhat et al. 2012; Shakeel et al. 2013; Noormohammadi et al. 2013; Zhang et al. 2013). Therefore, researchers could explore its application for authentication of traditional Chinese medicines. RAPD needs less DNA template and is relatively easy to handle, but is poor in reproducibility and stability, leading to restrictions in practical application. However, after converting RAPD markers into SCAR markers, the specificity and stability can be greatly improved, which makes it more convenient and efficient in the testing of different alleles (Dnyaneshwar et al. 2006; Li et al. 2010; Rajesh et al. 2013). Since they can identify a single or a few bands instead of a complex pattern, SCAR markers are more straightforward than other molecular techniques, such as RAPD, SSR, ISSR and AFLP.

In this study, we selected the clear and bright amplified DNA bands from RAPD markers for SCAR marker development. In SCAR, pairs of 18–25 bp oligonucleotide primers specific to the sequence of polymorphic bands can be used to amplify the characterized regions from genomic DNA under stringent conditions, which makes these markers more specific and dependable as compared to RAPD markers. Based on the sequences of our cloned DNA fragments, four SCAR primer pairs (LY2-1, LY4-7, LY4-8 and LY5-2) were designed. Genomic *D. longan* DNAs collected from 5 different regions within China, contained the cloned DNA fragments. As shown in Figures 4,5,6,7, the primers were generated from 250–360 bp bands in all *D. longan* cultivars, while no amplicon was observed in other species. Thus, the genetic polymorphism observed among the cultivars is interesting and can be used to develop markers for *D. longan*-specific identification.

Traditionally, *D. longan* fruit is used for several diseases (Park et al. 2010; Prasad et al. 2010; Zhong et al. 2010; Mei et al. 2013). Recently it was found that the dried longan seed extracts also have potential inhibitory effects on cancer cell invasion (Panyathep et al. 2013). However, *D. confinis*, a species of plant in the genus Dimocarpus family, with an oval-shaped drupe fruit similar to *D. longan* (http://www.people.com.cn/GB/paper503/13144/1179090.html), is very hard to distinguish from *D. longan* in the market only by morphology. If people sell *D. confinis* as longan, and someone purchase mistakenly and eat, they will experience vomiting, diarrhea and psychiatric disorder including anxiety, depression, insomnia, apprehension, auditory and visual hallucination, and torpid reaction. To distinguish the *D. longan* and *D.confinis*, PCR amplifications were performed by using above mentioned SCAR primers from Table 1. The results showed that the PCR products with expected size were observed only in *D. longan* samples by SCAR markers LY2-1, LY4-7 and LY5-2, without any amplification in *D. confinis* (Figure 8), which suggests that we have developed RAPD-SCAR markers successfully for identification of *D. longan* from the other species with similar morphology. Although the PCR product in *D. confinis* (LL) with same expected size from *D. longan* samples by SCAR marker Y4-8 were detected, which indicates that this marker cannot be used in the identification of *D. longan* from *D. confinis*, we can demonstrate that the DNA quality extracted from *D. confinis* is excellent*.* Therefore, our results showed that the developed SCAR markers could be useful to assess the genetic diversity and population structure of *D. longan* from *D. confinis*.

In this study, we obtained four clones by improved RAPD and DNA sequencing in *D. longan*, and developed stably diagnostic Sequence-Characterized Amplified Region (SCAR) markers for DNA fingerprinting to distinguish the fruit of *D. longan*, as a species of plant in the genus *Dimocarpus* with similarity to that of *D. confinis* with poisonous nature. Our results have shown that these SCAR markers could be very useful as a specific *D. longan* variety authentication, particularly for distinguishing *D. longan* from *D. confinis*.
